# An Exploratory Study of Honey Consumption Preferences: Insights from a Multi-Model Approach in Kosovo

**DOI:** 10.3390/foods15020334

**Published:** 2026-01-16

**Authors:** Arbenita Hasani, Oltjana Zoto, Manjola Kuliçi, Njomza Gashi, Salih Salihu

**Affiliations:** 1 Department of Food Technology with Biotechnology, Faculty of Agriculture and Veterinary, University of Prishtina, 10000 Prishtina, Kosovo; arbenita.hasani@uni-pr.edu (A.H.); njomza.gashi@uni-pr.edu (N.G.); 2 Department of Economy and Rural Development Policies, Faculty of Economy and Agribusiness, Agricultural University of Tirana, 1029 Tirana, Albania; ozoto@ubt.edu.al; 3 Department of Food and Biotechnology, Faculty of Biotechnology and Food, Agricultural University of Tirana, 1029 Tirana, Albania; mkulici@ubt.edu.al; 4 Center for Complex Systems and Microbiome Innovations, Faculty of Agricultural and Food Sciences and Environmental Management, University of Debrecen, 4032 Debrecen, Hungary

**Keywords:** honey, consumer behavior, willingness to pay, product attributes, storage practices

## Abstract

This study examines consumer behavior, preferences, and knowledge regarding honey in Kosovo to inform more effective production, marketing, and policy strategies. Data were collected from 503 respondents through an online questionnaire and analyzed using a combination of artificial neural networks (ANN), decision tree modeling (CHAID), and ordinal logistic regression. The results show a high prevalence of honey consumption, strong preference for locally produced honey, and significant variability in consumer willingness to pay (WTP) based on knowledge, income, and trusted information sources. ANN identified recommendations and product familiarity as primary predictors of WTP, while the decision tree highlighted knowledge and income as key variables for segmentation. The ordinal logistic regression confirmed the importance of perceived quality and product attributes, particularly botanical and geographical origin, in shaping purchasing decisions. The use of complementary statistical models enhanced both predictive power and interpretability. The findings highlight the crucial role of consumer education and trust cues in fostering sustainable honey markets in Kosovo.

## 1. Introduction

Beekeeping provides multisystemic benefits [1]. It generates goods and services that contribute to an increase in the per capita income of families, to the creation of jobs, both directly and indirectly, and to the sustainable development of rural areas [2,3,4,5,6]. Beekeeping also contributes to the conservation of natural biodiversity and minimizes pressure on forests [7,8]. Similarly, honey beekeeping produces essential, marketable, high-value products, including propolis, pollen, royal jelly, wax, and bee venom [1,6,9]. These products have several unique characteristics, depending on their botanical origin, such as local flora and nectar source [9,10], geographical origin [1,11], and beekeeping practices [12,13].

The beekeeping sector in Kosovo holds significant importance both economically and environmentally. With approximately 7500 beekeepers managing around 250,000 beehives, the sector not only generates income for thousands of rural families but also supports biodiversity and agricultural productivity through essential pollination services. Thus, honey production is a significant economic activity, as it impacts thousands of households in both rural and urban areas [1,14]. For that reason, research that evaluates consumer evaluation of the attributes related to the honey is necessary to adapt the production and pricing and, tailor the marketing to increase the economic value of the honey production activity Additionally, consumer-based knowledge will allow small producers to specialize and place the production by designing different marketing strategies in different segments [15]. Despite the growing international literature on honey attributes and consumer perceptions, empirical studies focusing specifically on honey consumption behavior in Kosovo remain very limited. Existing research in the country has largely examined preferences for domestic origin, traditional foods, and organic products, but has not explored honey-specific attributes, trust cues, or willingness to pay. Due to this lack of prior evidence, the present study adopts an exploratory approach aimed at identifying behavioral patterns and attribute trade-offs rather than testing predefined hypotheses. This approach is further justified by the absence of established models for analyzing honey consumption in the Kosovar context, making an exploratory design appropriate for uncovering underlying consumer dynamics.

Different researchers have focused on the attributes linked to honey, such as price, country of origin (COO), European origin-based quality schemes such as Protected Denomination of Origin (PDO), Protected Geographical Origin (PGI), production landscape, packaging, texture, production type (organic vs. conventional) [14,15,16,17,18,19,20,21,22]. The common basis of the findings of these studies is that honey is an example of a premium food product with enhanced quality properties, perceived by consumers as an important component in the human diet [16,23,24] and it has become more popular among consumers mainly due to its nutritional and medical benefits [23,24,25]. Honey is a functional food with health-beneficial properties and is already used as a medical device in wound care management [23]. It has proven effective against viral infections due to its potent antioxidant and anti-inflammatory properties, which attenuate oxidative damage induced by pathogens and enhance the immune system [26]. Although honey is considered a high-quality, premium food, it is more exposed to adulteration, mislabeling, and unethical mixing with other substances [27]. Moreover, the negative messages disseminated in the media regarding the international honey market and the COVID-19 pandemic situation also influenced consumer choices [28]. Therefore, honey adulteration is not the only factor impacting the economic losses that the sector is facing; it is also the difficulty in marketing this product. Similarly, its limited availability, proven curative properties, and heightened health concerns have raised the demand for the natural product [24,26,27]. In consequence, analyzing the trade-offs between honey attributes would be interesting. Despite these well-recognized health benefits, honey and other bee-derived products may also cause allergic reactions in individuals with sensitivities. Allergic reactions to honey and its products, though uncommon, can pose risks due to potential cross-reactivity with bee venom, particularly affecting people with atopy or lung conditions [29]. Honey can trigger anaphylaxis, generalized urticaria, or angioedema due to pollen proteins or bee salivary gland secretions, particularly in patients sensitized to aeroallergens like mugwort [30]. Acknowledging these potential allergic effects provides a more balanced perspective on consumer health perceptions and risk awareness.

Several studies conducted within the food sector in Kosovo have shown that origin is an important attribute in consumer choices, as it generates expectations of other attributes, such as better taste and safety [31,32,33,34]. Consumer studies on product attributes, such as “Made in Kosovo,” organic, and traditional products, indicate that consumers tend to associate domestic origin with safety, authenticity, and trustworthiness [32,34,35,36,37]. Similarly to findings in neighboring Albania, Kosovar consumers often perceive local products as fresher, tastier, and less industrialized due to the relatively low intensity of input use and small-scale production systems that characterize the country’s agriculture [38].

Previous studies on honey markets across countries such as Spain, Italy, Poland, Turkey, and Brazil have consistently highlighted the importance of attributes like botanical and geographical origin, organic certification, packaging, taste, and price in shaping consumer preferences and willingness to pay [17,39,40,41,42]. These findings have been widely applied in marketing strategies by local and international honey producers, who use origin labeling, certification schemes, premium packaging, and branding to differentiate their products and build consumer trust [43]. Research on local food markets similarly shows that consumers often associate domestic origin with authenticity, freshness, and safety, reinforcing the relevance of origin-based cues in purchasing decisions [31]. However, comparable studies focusing specifically on honey consumption behavior in Kosovo are scarce, underscoring the need for the present research to fill this gap and contextualize international insights within the local market. Kosovo represents a relatively underexplored yet promising context where the honey sector plays a significant role in rural livelihoods but faces structural weaknesses, including limited market organization, low consumer awareness of quality schemes, and insufficient institutional support [44]. Recent surveys highlight a strong consumer preference for local honey, primarily driven by trust in domestic producers and the absence of standardized certification and traceability systems. In this institutional context, informal cues such as producer reputation, origin, and word-of-mouth recommendations become central in shaping purchasing behavior. This study contributes to filling this research gap by analyzing honey consumption behavior in Kosovo, focusing on how socio-demographic characteristics, product-related knowledge, and attribute preferences influence consumers’ willingness to pay (WTP) for high-quality honey products. This study aims to provide a comprehensive analysis of honey consumption behavior in Kosovo by examining how socio-demographic characteristics, product-related knowledge, and attribute preferences influence consumers’ willingness to pay (WTP) for high-quality honey products. The specific objectives are

1. To explore socio-demographic influences on honey consumption patterns and WTP, focusing on variables such as age, gender, income, education level, family size, and area of residence (urban vs. rural).
2. To evaluate the role of subjective knowledge and perceived benefits related to honey and other bee-derived products, and how these shape satisfaction and usage frequency.
3. To identify the most valued honey attributes (e.g., botanical origin, geographical origin, organic certification, price, taste, packaging) and purchasing preferences (e.g., direct from a beekeeper, local market, pharmacy).
4. To assess the importance of information sources and informal trust networks, such as recommendations from doctors, pharmacists, friends, and family, in influencing WTP and product perception.
5. To segment consumers based on behavioral patterns using a multi-method analytical framework combining artificial neural networks (ANN), decision trees (CHAID), and ordinal logistic regression, to identify clusters with differentiated price sensitivities and motivations.

## 2. Materials and Methods

### 2.1. Data Collection

This study adopts a quantitative research approach to explore consumer behaviors, preferences, and honey-related knowledge in Kosovo. Using Yamane’s formula [45] for a population of 1.6 million, the required sample size was estimated at roughly 400 respondents; our achieved sample of 503 therefore provides sufficient statistical representativeness. The target population consisted of adult consumers (18 years and older) residing in Kosovo. Because no national database of honey consumers exists, the study employed a convenience sampling strategy. The questionnaire was distributed both online through social media platforms and in person using an intercept approach, where researchers approached individuals in public squares and other high-traffic areas and invited them to complete the survey. The study used a convenience sampling approach, distributing the questionnaire through social media and by directly approaching individuals in public squares and high-traffic areas to obtain a broad and diverse group of respondents. A total of 503 individuals accessed the questionnaire, all of whom completed it, and all responses were fully included in the analysis. Data collection was carried out between January and July 2024. The first section included socio-demographic variables such as age (coded 1 = 18–24, up to 5 = over 55), gender (1 = female, 2 = male), family size, residential location (1 = urban, 2 = rural), household income (1 = <200 EUR, up to 6 = >1500 EUR), and education level (1 = middle school, up to 5 = doctorate). The second section measured consumers’ knowledge about honey and related bee products on a 5-point Likert scale, ranging from 1 (no knowledge at all) to 5 (very knowledgeable). Respondents were also asked about their sources of information, selecting from options such as scientific articles, social media, doctors, friends, or pharmacists, with combinations coded accordingly. Perceived satisfaction with the health benefits of honey was measured using a satisfaction scale.

The third section assessed the influence of recommendations on purchasing behavior, identifying whether the recommendation came from doctors, pharmacists, family, friends, beekeepers, or others. Willingness to pay (WTP) for high-quality honey was captured via four options: EUR 5–EUR 15, EUR 15–EUR 25, over EUR 25, and no answer (4). To explore perceived quality, a follow-up question was asked whether consumers believe that the price justifies the quality of honey. Consumers were then asked to select the most important honey attributes influencing their purchase decisions, including botanical origin, geographical origin, price, organic certification, taste, producer/brand, consumer reviews, and labeling (each coded from 1 to 10). Purchasing preferences were evaluated through the preferred purchasing channel (e.g., beekeeper, market, pharmacy, online, etc.) and packaging (e.g., glass, plastic, inox, or other).

Additional questions explored consumption behavior and handling practices, including the time to consume after purchase (ranging from 3 months to more than 2 years) and seasonal consumption preferences (spring, summer, autumn, winter, all seasons, or no specific period). The questionnaire also inquired about the preferred type of honey (e.g., chestnut, acacia, meadow, mountain, pine) and the average annual consumption for each household member category (children, youth, parents, grandparents). Each categorical variable was numerically coded to allow for statistical modeling using advanced techniques, including decision trees (CHAID), artificial neural networks (ANN), and ordinal logistic regression. This coding system facilitated a robust behavioral segmentation analysis and enabled insights into the drivers of consumer trust, product evaluation, and price sensitivity.

Participants were informed about the voluntary nature of the survey and assured of the confidentiality and anonymity of their responses. They were explicitly notified that they could withdraw at any time without consequences. To safeguard privacy and comply with ethical standards, all data were collected and processed following the European Union’s General Data Protection Regulation [46].

### 2.2. Statistical Analysis

Data was analyzed using IBM SPSS Statistics (version 25; IBM Corp., Armonk, NY, USA) and RStudio (version 4.3.2; RStudio, Inc., Boston, MA, USA) to ensure a comprehensive and robust examination of consumer behavior. Descriptive statistics (frequencies, percentages, means) were used to summarize socio-demographic profiles and honey consumption patterns. A series of inferential statistical tests were employed to explore relationships among variables. Spearman’s rank-order correlation assessed associations between ordinal variables, and Chi-square tests evaluated relationships between categorical variables. To explore deeper behavioral drivers, three advanced modeling techniques were employed: artificial neural networks (ANN), specifically Multilayer Perceptron models, were used to predict willingness to pay (WTP) based on key attitudinal and demographic inputs. Variables were rescaled, and the model was trained and tested on a 70/30 split. A complementary classification tree provided an interpretable segmentation of WTP profiles. Finally, an ordinal logit model was estimated to determine the effect of predictors on consumers’ willingness-to-pay (WTP) levels. This multi-method strategy enabled the assessment of predictive power (via artificial neural networks), interpretability (via decision trees), and inferential robustness (via ordinal regression), providing a comprehensive, hybrid analysis of consumer preferences and willingness-to-pay.

### 2.3. Respondents’ Characteristics

From a total of 503 respondents, the survey collected valuable data on various socio-demographic characteristics (Table 1).

In terms of gender, 60.2% of the participants were male, while 39.8% were female. Regarding age, the largest group was between 25 and 34 years (40.4%), followed by 18–24 years (16.9%) and 35–44 years (24.7%). Regarding education, most respondents held a Bachelor’s degree (40.6%), followed by those with a high school education (28.4%) and a Master’s degree (21.7%). A smaller proportion had a Doctorate or completed middle school. In terms of income, the most significant proportion of respondents earned between EUR 600 and 1000 (23.3%), followed by those earning between EUR 1000 and 1500 (20.9%), and those earning above EUR 1500 (31.0%). A smaller number had income levels below EUR 600.

## 3. Results

### 3.1. Overview of Honey Consumption and Purchasing Preferences

**Figure 1:**
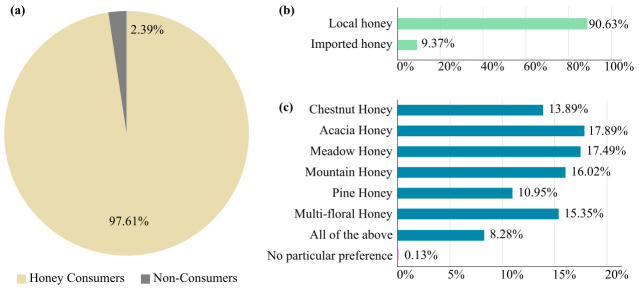
Honey consumption preferences: (**a**) Pie chart illustrates the level of honey consumption, (**b**) bar chart displaying purchasing preferences for local versus imported honey (**c**) bar charts displaying the most preferred types of honey.

Most respondents (97.61%) reported consuming honey (Figure 1a), reflecting its widespread presence in Kosovar households. Among them, 90.63% expressed a preference for local honey over imported alternatives (Figure 1b), underscoring a high level of trust in domestic producers and a strong inclination toward locally sourced products. Participants associated local honey with freshness, perceived quality, and authenticity. This preference is further reinforced by the fact that many consumers reported using traditional methods to verify authenticity, including visual inspection, taste testing, heat exposure, and topical application.

Regarding honey types, Acacia, Meadow, and Mountain honeys were the most preferred varieties (Figure 1c). These types are valued for their flavor, texture, and perceived health benefits. Pine honey was the least consumed type, possibly due to lower availability or lower familiarity. Interestingly, 8.28% of participants reported consuming all types of honey, suggesting openness to variety and experimentation.

**Figure 2:**
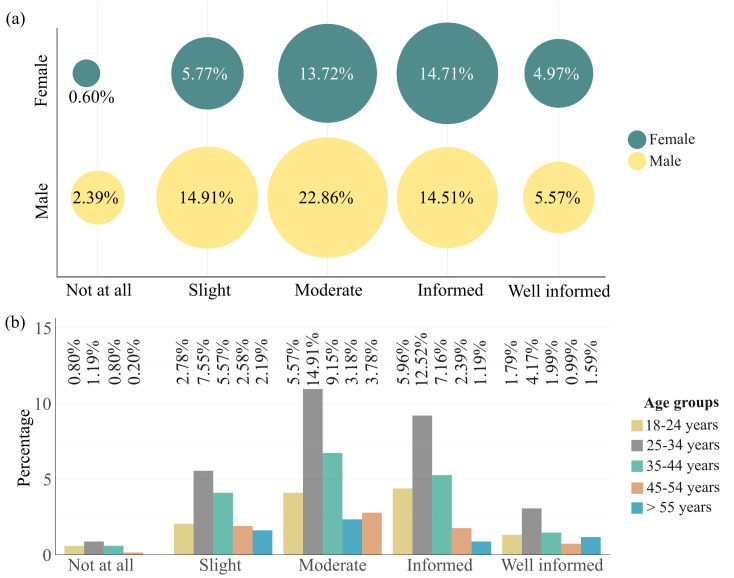
Consumer knowledge levels about honey by gender and age group. Source: Author’s elaboration. The top panel (**a**) shows the distribution of self-reported knowledge levels among males and females, represented as bubble plots. The bottom panel (**b**) presents a bar chart illustrating the differences in knowledge level across age groups. Knowledge is categorized into five levels: not at all, slight, moderate, informed, and well informed. Results indicate higher knowledge concentration among individuals aged 25–34 and among males reporting moderate knowledge. Note: Percentages represent the share of respondents within each gender and age category who self-identified with the respective knowledge level.

Figure 2 illustrates purchasing channels by gender. Direct purchase from beekeepers was the most preferred source, particularly among males (41.76%) and to a lesser extent among females (28.22%). Open markets were also popular, especially among females (8.97% vs. 5.38% males). Supermarkets, pharmacies, and online stores were less commonly chosen, indicating a general preference for direct, traceable, and trust-based sources.

Lastly, a Chi-square analysis revealed significant associations between reasons for honey consumption and various demographic factors (*p* < 0.05). Males were more likely to consume honey for health benefits (27.09%) and nutritional value (18.10%), whereas females reported lower levels of these motivations. Age also played a significant role: individuals aged 25–34 cited health and nutrition more often, while younger respondents (18–24) showed a broader distribution across health, taste, and sweetness. Education had a significant influence: higher levels of education were associated with a greater likelihood of reporting health-related motivations. In contrast, those with lower education levels were more frequently selected “all reasons” or “taste/sweetness.” Additionally, higher income and urban residence were associated with a stronger orientation toward health and nutrition as primary motives for honey consumption.

### 3.2. Variation in Honey Use from Infancy to Adulthood

Honey consumption in Kosovo shows a clear age-related pattern, increasing progressively from infancy through childhood and adolescence to adulthood (Table 2). Infants aged 0–1 year show low consumption rates, with 18.1% reporting zero use and only small percentages in higher categories. However, 14.9% of respondents reported some level of honey intake among infants, despite dietary guidelines advising against it. Alarmingly, 48.5% of participants were unaware of the risks of infant botulism, and only 14.1% reported high awareness see Table 2.

From age one onward, honey consumption steadily increases. In the 5–12-year group, 15.3% consume 1–2 kg per year; in adults aged 18 years and older, 28.4% report this level, and an additional 14.5% consume 3–4 kg annually. Parents and grandparents also report meaningful consumption levels, though older adults tend to consume more moderately. Across all groups, the most common intake levels are between 0.5 and 0.9 kg and 1 and 2 kg.

### 3.3. Consumption, Knowledge of Honey and Demographics

While age emerged as a strong determinant of honey consumption patterns, with clear increases observed from early childhood through adulthood, further analysis was needed to understand the role of broader sociodemographic characteristics and knowledge levels in shaping this behavior. The following section examines whether factors such as income, education, and place of residence influence honey consumption and how knowledge of honey’s health properties varies across demographic groups.

#### 3.3.1. Consumption Levels and Demographics

Statistical analyses revealed no significant associations between honey consumption levels and key sociodemographic factors, including education, income, and type of residence. Spearman’s rank correlation coefficients indicated weak, non-significant associations with education level (ρ = −0.038, *p* = 0.401) and income (ρ = 0.025, *p* = 0.572). Similarly, the Mann–Whitney U test showed no significant difference in honey consumption by place of residence (U = 16,909, *p* = 0.766). These findings suggest that while consumption increases with age, it is not significantly influenced by education level, income or urban versus rural residency. Instead, age appears to be the primary driver of honey use.

#### 3.3.2. Consumer Knowledge and Sociodemographic Differences

Although statistical tests showed no significant relationship between honey consumption and sociodemographic factors such as education, income, or place of residence, notable differences emerged in consumer knowledge levels across demographic groups. Overall, most respondents reported moderate knowledge about honey; however, significant variation was observed by gender, age, and educational level. Gender differences were statistically significant (χ^2^ = 17.046, *p* = 0.002), with men more frequently reporting moderate or slight knowledge compared to women (Figure 2a), which may indicate differences in access to or interest in nutrition-related information. Age also played a role: the 25–34 age group demonstrated the highest proportion of informed consumers, while knowledge declined notably among participants aged 45–54 (Figure 2b), suggesting a possible generational gap in information access or health engagement. Education level was positively correlated with knowledge (Spearman’s ρ = 0.3, *p* < 0.01), as university-educated individuals were more likely to report being “informed” or “well-informed”. These results suggest that while honey consumption is widespread across social groups, understanding of its health properties is more strongly influenced by educational attainment, age, and gender than actual consumption behavior.

Age also played a role: the 25–34 age group demonstrated the highest proportion of informed consumers, while knowledge declined notably among participants aged 45–54 (Figure 2b), suggesting a possible generational gap in information access or health engagement. Education level was positively correlated with knowledge (Spearman’s ρ = 0.3, *p* < 0.01), as university-educated individuals were more likely to report being “informed” or “well-informed”. These results suggest that while honey consumption is widespread across social groups, understanding of its health properties is more strongly influenced by educational attainment, age, and gender than actual consumption behavior.

### 3.4. Timing and Seasonality of Use

**Figure 3:**
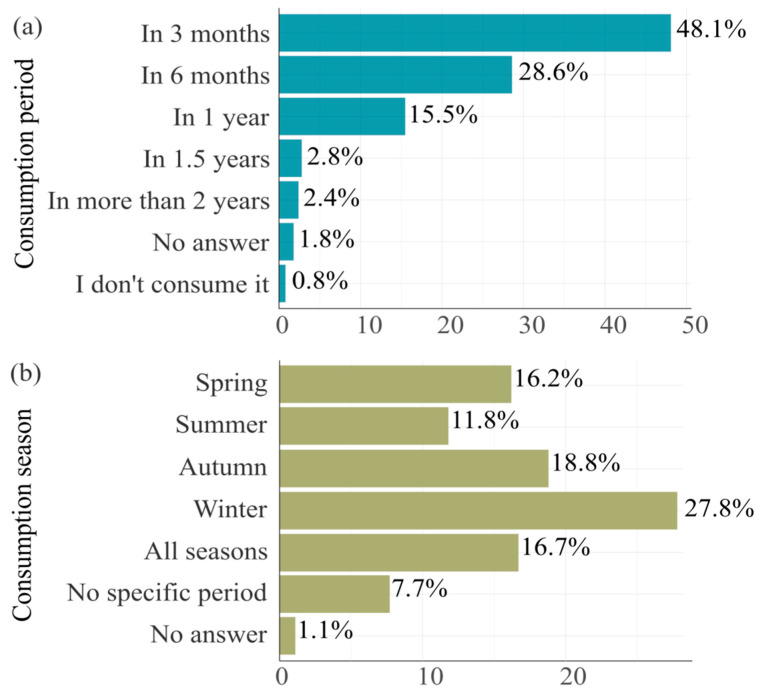
Timeframe of honey consumption after purchase (**a**) and seasonal preferences for honey consumption (**b**). Source: Author’s elaboration.

Honey consumption in Kosovo follows clear temporal and seasonal patterns. Nearly half of respondents (48.1%) reported consuming honey within three months of purchase, while another 28.6% did so within six months (Figure 3a). Only 15.5% extended consumption to one year, and usage declined sharply thereafter. These findings suggest that honey is used relatively quickly, likely due to its role in daily health routines or seasonal needs.

Seasonal preference data further support this interpretation. Winter was the most common season for honey consumption, with 27.8% of respondents indicating increased use during this time (Figure 3b). Autumn followed with 18.8%, possibly reflecting preparation for the colder months. Spring and summer saw moderate use, while 16.7% of respondents reported consistently consuming honey across all seasons. These trends reinforce the perception of honey as a functional food, particularly valued during times of heightened illness or seasonal transition.

**Figure 4:**
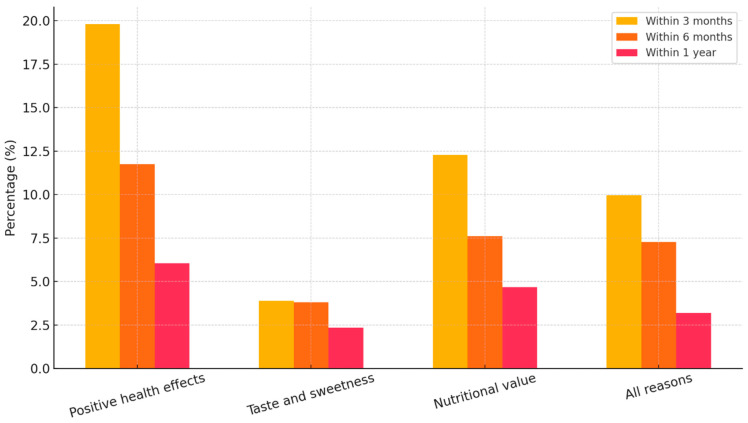
Honey consumption timing by motivation. Source: Authors’ elaboration.

This short-term consumption pattern aligns closely with the motives reported by respondents, see Figure 4. Those who consume honey for positive health effects are the most prominent group across all timeframes, particularly within the first three months (19.8%) and six months (11.8%). Similarly, participants who cited nutritional value or a combination of reasons (health, taste, and nutrition) also demonstrated higher usage in shorter periods. In contrast, those consuming honey primarily for flavor and sweetness were less represented in short-term usage brackets, implying that health-related motivations are a primary driver of quicker consumption. Although the Chi-square test for reason of consumption and period was not statistically significant (*p* = 0.304), the descriptive patterns suggest a strong behavioral tendency to consume honey rapidly when its use is perceived to serve a functional or therapeutic purpose, see Figure 4.

Seasonal preferences further reinforce this interpretation (Figure 3). Winter is the most common season for honey consumption (27.8%), followed by autumn (18.8%), with usage often linked to seasonal illnesses or immunity-boosting routines. A notable 16.7% of respondents reported consuming honey year-round, indicating a group of regular users who view honey as part of their ongoing health management. These findings highlight the functional framing of honey in consumers’ minds: not merely as a sweetener or traditional food, but as a health-enhancing product with seasonally heightened relevance.

Although seasonal honey consumption patterns were generally consistent across the population, a few notable differences emerged by demographic group. Residence type was the only demographic variable showing a statistically significant association with seasonal use patterns (χ^2^ = 17.252, *p* = 0.008). Urban residents reported higher honey consumption across all seasons, particularly in winter (23.1%) and autumn (15.3%), compared to their rural counterparts, who reported substantially lower rates during the same periods. This difference may reflect greater access to bee products in urban retail markets, increased exposure to health promotion campaigns, or differences in cultural practices related to seasonal health.

### 3.5. Information Sources and Product Attributes Influencing Honey Purchase

**Figure 5:**
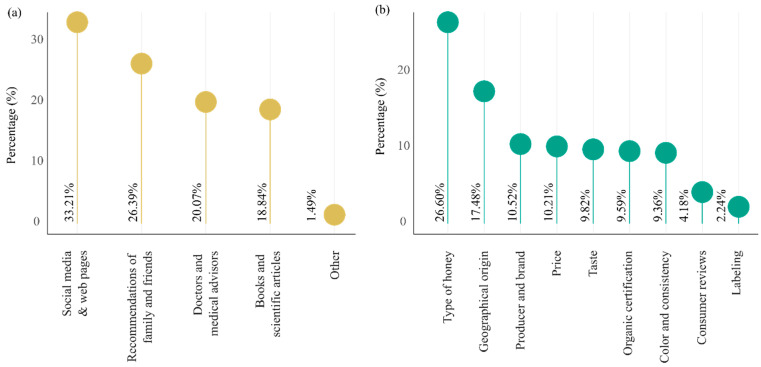
Sources of information and product attributes influencing honey purchasing decisions. (**a**) Sources of information influencing consumer decisions about honey. (**b**) Product attributes considered important when purchasing honey. Source: Author’s elaboration.

A combination of information channels and product-related factors shapes consumers’ decisions regarding honey purchases. As shown in Figure 5a, the most frequently cited sources of information are social media and online platforms, with 33.21% of respondents reporting reliance on websites, social media, and digital content for guidance. These digital sources are followed by interpersonal recommendations, including advice from family, friends, and medical professionals, which collectively represent a substantial influence. Scientific literature and books were mentioned less frequently but still contribute to knowledge acquisition, particularly among more informed consumers.

When considering the attributes that influence purchasing decisions, respondents emphasized the botanical origin (type of honey) and geographical origin as the most important product characteristics (Figure 5b). These preferences suggest a strong consumer interest in traceability and authenticity, as well as perceived quality based on floral and regional specificity. Additional influential factors include the producer or brand reputation, price, and taste, reflecting a balance between quality assurance and economic considerations. While organic certification and consumer reviews also played a role, labeling was the least important factor for most respondents.

### 3.6. Willingness to Pay for a High-Quality Honey Product

**Figure 6:**
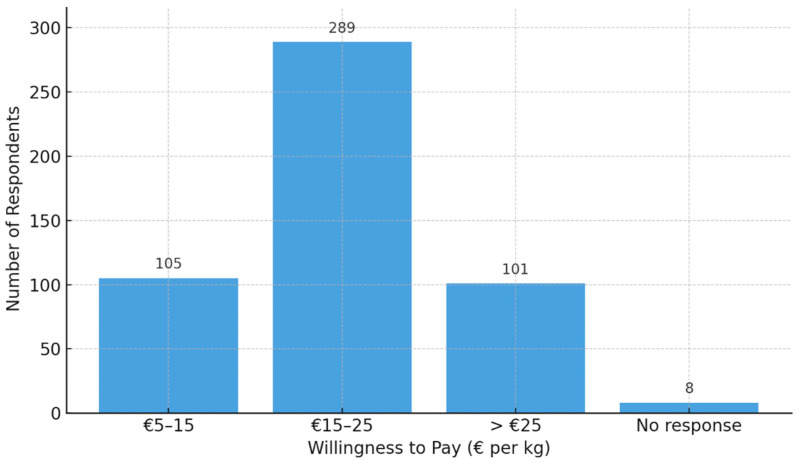
Willingness to pay for Honey (N = 503). Source: Author elaboration.

Survey findings indicate a clear preference among consumers for mid-range pricing when purchasing high-quality honey (Figure 6). Over half of respondents (57.5%) reported a willingness to pay between EUR 15 and EUR 25 per kilogram, while 20.9% preferred a lower price range of EUR 5 to EUR 15. Notably, a significant portion (20.1%) expressed a willingness to pay more than EUR 25, suggesting the existence of a premium consumer segment. Only a small percentage (1.6%) chose not to respond. These results demonstrate broad acceptance of moderate-to-high price points, highlighting the market potential for both standard and premium honey products.

The observed distribution of willingness to pay reflects a market primarily oriented towards perceived value and product quality. The majority of respondents fall within the EUR 15–25 range, supporting earlier findings in this study where consumers emphasized trust in local producers, health-related motivations, and preference for specific honey types [16,47,48,49]. One in five consumers is willing to pay more than EUR 25 per kilogram, which reinforces the presence of a quality-driven, possibly knowledge-rich segment, as also suggested by the ANN results, in which higher knowledge and trusted recommendations were strong predictors of WTP.

#### 3.6.1. Predicting Willingness to Pay Using ANN Modeling

To identify the key factors influencing consumers’ willingness to pay (WTP) for honey, an Artificial Neural Network (ANN) model was developed using a multilayer perceptron architecture with one hidden layer. The dependent variable was WTP per kg of honey, categorized into four price levels, while a combination of behavioral, knowledge-based, and demographic variables served as predictors. The dataset included 503 observations, of which 239 were valid and used for training and testing after accounting for missing values. The model achieved a training accuracy of 71.2% and a testing accuracy of 55.1%, indicating moderate predictive performance. The cross-entropy error was 136.736 for the training set and 71.970 for the testing set, with error stabilization after one consecutive step.

**Figure 7:**
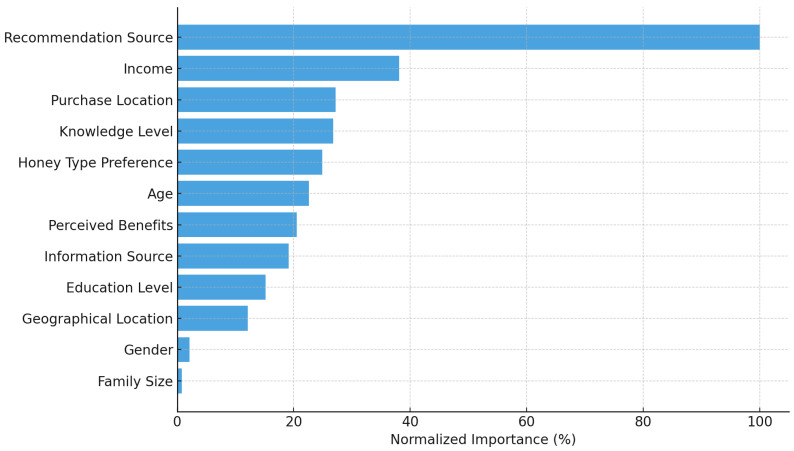
Variable importance from the ANN model predicting WTP. Source: Author’s elaboration.

The classification results (Figure 7) show that the model predicted mid-range WTP (category 2) with the highest accuracy, achieving a 97.2% correct classification rate on the test set. However, prediction accuracy was much lower for other categories, particularly the lowest and highest WTP segments, which were misclassified mainly. Analysis of independent variable importance revealed that the strongest predictor of WTP was whether someone had recommended the product to the respondent (normalized importance = 100%), followed by purchase location preference (27.2%), knowledge level about bee products (26.8%), and income level (38.1%). Other important factors included preferred honey type (24.9%) and perceived benefits from product use (20.5%). Demographic variables, such as gender, family size, and residential area, had a minimal influence on WTP predictions.

**Figure 8:**
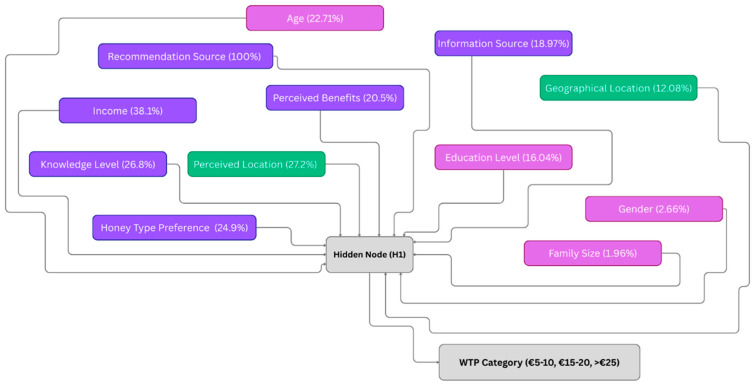
ANN structure for WTP prediction. Source: Authors’ elaboration.

Figure 8 illustrates the structure of the Artificial Neural Network (ANN) used to predict consumer willingness to pay (WTP) for high-quality honey. The model takes into account 12 input variables, including behavioral attributes (e.g., purchase location, recommendation source), product preferences, and demographic characteristics. These inputs are processed through a single hidden node (H1), which captures the nonlinear interactions among variables. The output layer categorizes WTP into four distinct classes: 1 = EUR 5–15, 2 = EUR 15–25, 3 = >EUR 25, 4 = No response. The ANN was trained to learn from consumer data patterns and estimate the most likely WTP category. Arrows represent the flow of information and weighted connections between layers, as learned during model training.

#### 3.6.2. Decision Tree Modeling: Interpreting Willingness to Pay Patterns

**Figure 9:**
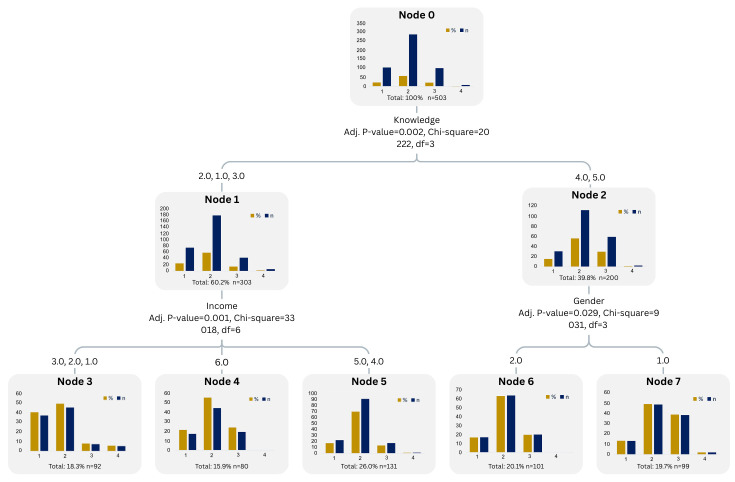
CHAID decision tree model showing how knowledge, income, and gender influence WTP categories for honey. Source: Author’s elaboration.

A decision tree model, utilizing the CHAID algorithm, was employed to investigate the impact of consumer characteristics on willingness to pay (WTP) for honey (Figure 9). The model included 503 respondents and utilized 11 predictors, with Knowledge, Household Income, and Gender emerging as statistically significant variables that shaped WTP categories. The root node indicates that most consumers (57.5%) fall into the mid-range WTP category (EUR 15–25), followed by lower (EUR 5–15, 20.9%) and higher (>EUR 25 EUR 25, 20.1%) WTP segments. The first and most influential split was based on knowledge level: respondents with high knowledge (scores 4–5) were assigned to Node 2, whereas those with low to moderate knowledge (scores 1–3) were assigned to Node 1. Among respondents with low to moderate knowledge (Node 1), Household Income further segmented WTP behavior. For example, Node 3 includes consumers with low income, where a higher proportion (39.1%) are willing to pay only EUR 5–15. Conversely, Node 5 represents high-income respondents, where 68.7% are willing to pay EUR 15–25, and 10.7% are willing to pay over EUR 25, indicating a higher perceived value for honey products. For high-knowledge respondents (Node 2), Gender became the next relevant split. Male respondents (Node 7) showed slightly higher interest in paying premium prices (38.4% > EUR 25) compared to females (Node 6), who were mostly clustered in the EUR 15–25 range (63.4%).

Further descriptive analysis of Node 7, representing high-knowledge male consumers, provides additional insight into the characteristics underlying their higher willingness to pay. Within this subgroup, respondents were predominantly employed or self-employed, with a notable representation of professionals and skilled workers, suggesting stable income sources and higher purchasing autonomy.

In terms of information acquisition, Node 7 consumers relied primarily on professional and expert-based sources, particularly recommendations from doctors, pharmacists, and beekeepers, rather than informal social networks or mixed information channels. This pattern reinforces the importance of perceived expertise and credibility in shaping premium valuation.

When purchasing honey, respondents in this node placed greater emphasis on intrinsic and credence attributes, especially botanical origin, geographical provenance, and perceived health benefits, while price sensitivity was comparatively lower. Taste and labeling played a secondary role, consistent with a functional and quality-oriented consumption logic.

Preferred purchasing channels among Node 7 consumers were direct sales from beekeepers and specialized outlets, rather than general markets or online platforms, indicating a preference for traceability, trust-based transactions, and direct producer–consumer relationships.

Overall, the decision tree reinforces the role of knowledge and income as key enabling factors and highlights the nuanced role gender plays when consumers are already informed. The final model comprised 8 nodes, a depth of 2, and a risk estimate of 0.425, indicating a moderate fit. The tree structure provides an intuitive way to segment honey consumers and target outreach or pricing strategies accordingly.

#### 3.6.3. Ordinal Logistic Regression Analysis

To address the limitations of the ANN and decision tree models, particularly their class imbalance and low prediction accuracy for extreme WTP categories, an ordinal logistic regression was conducted using the Logit link function (Table 3). The model demonstrated a strong fit and robustness in estimating the ordered categories of willingness to pay for high-quality honey (WTP). The model improvement over the null model was statistically significant (χ^2^ = 122.64, df = 47, *p* < 0.001), and goodness-of-fit indicators supported the model’s adequacy (Pearson’s *p* = 0.995, Deviance’s *p* = 1.000).

The model’s explanatory power was moderate to strong, with Nagelkerke R^2^ = 0.60, indicating that approximately 60% of the variance in WTP could be explained by the predictor variables. Among all predictors, income was the most statistically significant socio-demographic factor (β = 0.430, *p* = 0.006), indicating that respondents with higher incomes were more likely to be willing to pay a higher price for honey.

In terms of attitudinal and behavioral variables, the combined use of information sources from scientific articles and family/friends (Info_source: 1, 4) was negatively associated with WTP (β = −7.522, p = 0.008), suggesting that conflicting or diluted messaging may occur when traditional and research-based sources coexist. Knowledge levels showed a positive but non-significant trend toward higher WTP for those reporting “Good” (*p* = 0.128) or “Little” (*p* = 0.257) knowledge about honey, supporting the ANN and CHAID models, though without reaching statistical significance in this model.

In terms of attitudinal and behavioral variables, the combined use of information sources from scientific articles and family/friends (Info_source: 1, 4) was negatively associated with WTP (β = −7.522, p = 0.008), suggesting that conflicting or diluted messaging may occur when traditional and research-based sources coexist. Knowledge levels showed a positive but non-significant trend toward higher WTP among those reporting “Good” (*p* = 0.128) or “Little” (*p* = 0.257) knowledge of honey, supporting the ANN and CHAID models. However, the association did not reach statistical significance.

It should be noted that the three modeling approaches serve distinct analytical purposes. The ANN model is used as an exploratory tool to identify nonlinear relationships and rank variable importance. In contrast, the CHAID decision tree primarily serves as a validation and interpretability mechanism, translating complex patterns into transparent segmentation rules. Ordinal logistic regression serves as the inferential component of the analysis, enabling formal hypothesis testing and explaining variance using pseudo-R^2^ measures. As pseudo-R^2^ indices are meaningful only for likelihood-based models, they are reported exclusively for the ordinal regression, whereas predictive accuracy and risk estimates are used for the machine learning models.

## 4. Discussion

### 4.1. Trust, Preferences, and Demographic Influences in Honey Purchasing

The findings confirm that honey is a culturally embedded and highly valued product in Kosovo, consumed by nearly all respondents. The strong preference for local honey aligns with earlier results on consumer motivations, particularly trust, authenticity, and quality assurance [16]. This mirrors patterns observed across Southern and Eastern Europe, where local sourcing is often linked with naturalness and food sovereignty [31,50,51,52,53,54]. Similarly, preferences build upon the earlier findings on the role of botanical and geographical origin in consumer evaluations (Section 3.5), showing that perceived purity, traceability, and health benefits are deeply interwoven in honey-related behavior [55].

The choice of honey types further illustrates how consumers associate functional benefits with flavor diversity, reinforcing the earlier result that health, rather than just taste, is a core driver of honey use. The popularity of Acacia and Mountain honey varieties suggests a shared cultural and sensory preference, as well as possible alignment with widely held beliefs about their therapeutic effects. The type of honey is frequently associated with its curative properties, especially among consumers living in mountainous areas, where the local flora where bees forage is considered essential for determining honey quality. These perceptions are often supported by collective knowledge passed down through generations. This is further evidenced by the primary health-related motivation for honey consumption in these regions.

Gender, age, and education differences offer additional nuance to the discussion of honey motivations. Male and higher-income respondents tend to emphasize health and nutrition, suggesting more purpose-driven consumption, while women and younger respondents show more diversified motivations, possibly shaped by family traditions or broader exposure to product types. These insights complement earlier findings on knowledge and information sources, where more educated consumers reported higher levels of knowledge and were more responsive to digital or interpersonal recommendations. Together, these results present a holistic picture: consumer behavior around honey in Kosovo is driven by a mix of trust, tradition, and health consciousness, shaped by personal experience, information exposure, and socio-demographic background. The preference for local, traceable, and health-related honey is evident throughout the dataset, underscoring the importance of supporting local producers, clearly communicating authenticity, and engaging consumers through trusted and informed channels.

The results highlight the widespread use of honey in Kosovo, with consumption patterns closely aligned with age and dietary autonomy. Low intake among infants largely reflects adherence to health guidelines, though the presence of consumption in this group and limited awareness of infant botulism signal potential risks. As age increases, honey becomes a more regular component of the diet, especially among adults, suggesting a link to health-conscious behavior and traditional culinary practices. The predominance of moderate consumption levels (0.5–2 kg) across age groups suggests that honey is integrated into daily diets, albeit not in excessive quantities. These findings highlight the importance of targeted health education in promoting awareness about the safe use of honey, particularly among caregivers of young children.

### 4.2. Timing and Seasonality of Honey Consumption

The findings reveal that honey is predominantly consumed shortly after purchase and exhibits high seasonal variation, with winter the most prominent period of consumption. This suggests that honey is perceived not merely as a food item but as a functional product closely tied to health maintenance, particularly during periods of increased vulnerability to illness, such as the cold and flu season. The concentration of consumption in autumn also implies a preventive behavioral pattern, where individuals may increase honey intake to prepare for the colder months. The fact that a significant share of respondents (16.7%) reported consuming honey throughout the year indicates that for a portion of the population, honey has transcended seasonal use and become a regular part of their dietary routine, likely influenced by health-conscious practices.

These findings align with previous studies showing that honey consumption increased significantly during winter months, primarily driven by beliefs in its immune-boosting properties [56,57]. They observed that health-related motivations, including the use of natural remedies and seeking cough relief, were primary drivers of winter honey consumption. However, more continuous, year-round use was also associated with higher income and health consciousness. Compared to these studies, the Kosovo results follow a similar seasonal pattern, reinforcing the idea that honey serves not only culinary but also preventive and therapeutic roles in consumer behavior. The relatively fast consumption timeframe (within 3 to 6 months for over 75% of users) further supports this functional interpretation. This may also reflect purchasing habits that prioritize freshness, local sourcing, or specific health needs rather than long-term storage. Overall, these patterns highlight the importance of understanding honey not only as a traditional or cultural product but also as a health-related commodity influenced by seasonal and temporal dynamics.

### 4.3. Information and Attribute Drivers of Honey Purchasing

These results underscore the growing importance of digital information ecosystems in shaping consumer behavior toward natural products such as honey. The prominence of social media and web-based content suggests that consumers, particularly younger and tech-savvy demographics, are increasingly reliant on rapid, accessible, and visually engaging sources of information [58]. This trend is consistent with findings in recent studies, which highlight the role of digital communication in promoting food-related health awareness and influencing purchasing behavior. However, the continued relevance of interpersonal and professional recommendations indicates that trust-based, offline channels remain influential, particularly for health-related products. In both Albania and Kosovo, honey is often purchased through informal family contacts or offline social networks. This behavior reflects the fact that honey is perceived as a “black box” product, where consumers cannot easily verify quality and thus rely heavily on trust in the seller as a key safety mechanism [59].

The high importance placed on botanical and geographical origin reflects a broader consumer shift toward product authenticity, provenance, and perceived purity [60]. This is well aligned with European market trends, where origin-specific and monofloral honeys are often considered indicators of higher quality and safety [61,62,63,64]. Studies such as Kokthi et al. [65] have shown that consumers associate origin with trust and are willing to pay a premium for locally sourced or geographically indicated products. Interestingly, while price and taste remain relevant, their secondary position in comparison to origin and type highlights that consumers may be prioritizing perceived health value and authenticity over sensory or cost factors. The relatively low importance of labeling suggests a potential gap in consumer understanding of what labels convey, or perhaps a mistrust in labeling standards, a finding that aligns with previous concerns in food labeling literature [31,65,66].

Overall, these patterns suggest that marketing strategies and public information campaigns for honey should emphasize origin transparency, the health-related benefits of botanical types, and engage effectively through digital communication channels, while not overlooking the credibility conferred by professional and familial recommendations.

### 4.4. Predicting Willingness to Pay Using ANN Modeling

The ANN model generated valuable and interpretable insights into the drivers of willingness to pay for honey, despite its varying classification performance across price segments. Its high accuracy in the EUR 15–25 segment, which represents most consumers, demonstrates the model’s value in identifying dominant behavioral patterns in a typical mid-range pricing context.

However, its limited accuracy in predicting the lower and upper WTP categories, likely due to class imbalance, underscores a common limitation of ANN models working with skewed categorical data. Despite this, the model’s variable importance provides valuable guidance: social influence, purchase behavior, and knowledge emerge as critical factors shaping price decisions, reinforcing earlier findings in this study on trust, local preference, and product familiarity. While ANN models are often criticized for being “black boxes,” their ability to highlight nonlinear relationships and variable influence is beneficial when studying complex consumer behavior. In conclusion, the ANN model, while not without limitations, offers a robust foundation for understanding core WTP drivers and supports the strategic segmentation of honey consumers based on behavioral profiles and informational exposure.

### 4.5. Decision Tree Modeling-Interpreting Willingness to Pay Patterns

The decision tree model provides valuable, interpretable insights into the key factors influencing consumers’ willingness to pay (WTP) for high-quality honey. By identifying knowledge, income, and gender as the most discriminating variables, the model aligns with previous findings in the food behavior literature that emphasize the importance of informational and socioeconomic factors in shaping consumer valuations [47,59]. Notably, knowledge about honey and its health benefits emerged as the primary splitter in the model. This is consistent with studies showing that informed consumers are more likely to perceive added value in food products and are thus more willing to pay a premium [67]. The strong influence of household income on the WTP decision further supports evidence that income enables not only purchasing power but also access to differentiated, high-quality products [68]. Informed, high-income respondents in this study were more likely to choose premium price points for honey, reinforcing the dual importance of capacity and awareness in food valuation. The model also reveals a nuanced role for gender within high-knowledge groups. Male respondents were more likely than females to select premium WTP categories. While gender effects in WTP studies are often inconsistent, some studies suggest that men may exhibit greater willingness to pay when the product is associated with performance or quality attributes [69], which may be the case for honey, which is often viewed as a functional or health-enhancing product.

The deeper profiling of Node 7 enhances the interpretability of the decision tree results and provides actionable insights for premium honey positioning. High-knowledge male consumers with elevated willingness to pay appear to combine purchasing capacity with strong reliance on expert-driven information and intrinsic quality cues. Their preference for direct purchasing channels and origin-based attributes suggests that premium valuation is closely linked to trust, perceived authenticity, and functional health benefits rather than branding or price promotions. This finding supports the role of targeted communication strategies that emphasize scientific credibility, producer transparency, and product provenance when addressing high-value consumer segments in emerging honey markets.

The decision tree model (CHAID) proved valuable for interpreting the factors influencing willingness to pay (WTP) for honey. Its transparent structure revealed that knowledge, income, and gender were the most significant predictors of consumer price sensitivity. This aligns with previous research, which shows that higher product knowledge and purchasing power increase the likelihood of consumers paying for quality attributes [67,68]. The tree’s strength lies in its interpretability and practical segmentation. It clearly outlines how combinations of consumer traits lead to different WTP categories, making it a valuable tool for targeting communication, pricing, or education strategies [70]. Compared to the ANN model, the decision tree offers a more accessible framework for non-technical audiences while confirming key predictors.

However, the model is limited in its predictive flexibility. Due to class imbalance, it disproportionately favored the majority group (EUR 15–25 WTP) and failed to capture variation in premium or budget segments. Moreover, while 11 variables were tested, only three were retained in the final splits, suggesting either dominant effects or CHAID’s lower sensitivity to complex interactions. However, studies analyzing factors influencing WTP for honey in various countries (Serbia, China, Korea) often use classification and regression tree (CART) methods or similar to segment consumers by demographics, attitudes, and identify key predictors of WTP [21,700^71^71]. The decision tree reveals that consumer knowledge is the primary driver of willingness to pay for honey. When knowledge is limited, income constraints dominate price decisions, whereas at higher knowledge levels, socio-demographic factors such as gender shape willingness to pay, suggesting a shift from budget-driven to value-driven decision-making. In summary, the decision tree provides practical insights and clarity of communication. However, its limitations in capturing minority behaviors support the use of hybrid approaches, combining interpretable models with predictive techniques like ANN for robust behavioral research.

### 4.6. The Ordinal Regression Model

The ordinal regression model provides a more statistically robust perspective on the factors influencing consumers’ willingness to pay (WTP) for honey. It confirms prior findings from the ANN and CHAID models, which indicate that income and knowledge are central drivers, with income emerging as the only statistically significant individual factor across all models. The result aligns with previous literature, which consistently shows that income predicts premium food choices and health-oriented purchases [32,67].

The negative coefficient for combined information sources (scientific + family/friends) may reflect conflicting credibility judgments or confusion introduced by overexposure to mixed messages, a theme echoed in trust and health behavior research [72]. Suggesting that while education campaigns can raise awareness, overly fragmented channels may dilute effectiveness. This nuanced result underscores the importance of developing coherent, targeted communication strategies, preferably grounded in credible sources and tailored to individual income levels and information literacy. Importantly, the ordinal regression model complements machine learning approaches by providing more precise parameter estimates and significance testing, which is particularly useful for policy and communication design.

While artificial neural networks (ANN) and decision tree models such as CHAID are primarily designed for prediction, their moderate accuracy, 71.2% for ANN and 57.5% for CHAID in this study, does not diminish their value in exploratory analysis. Many studies demonstrate that even when predictive power is modest, these models serve as powerful tools for uncovering latent structures, nonlinear relationships, and relevant segmentation rules that can inform subsequent inferential analysis [73,74]. Machine learning methods are increasingly used not only for endpoint prediction but also as intermediate steps that generate hypotheses, classify respondents into meaningful groups, or identify influential variables and functions, which are particularly useful in behavioral consumer studies [73,74,75,76]. In this study, the ANN highlighted complex, nonlinear interactions among variables, while CHAID clarified how knowledge and income jointly segment willingness-to-pay (WTP) profiles.

Subsequently, the findings from these models were validated and extended using ordinal logistic regression. The model demonstrated a robust fit, with a Nagelkerke R^2^ of 0.60, indicating that knowledge, income, and specific informational cues (e.g., doctor recommendations) significantly influence willingness to pay (WTP). This integration of exploratory machine learning with inferential modeling exemplifies a recommended multi-method approach in contemporary empirical research [76]. This multi-method approach strengthens the validity of the results. Strengthens both the interpretability and generalizability of results, especially in emerging markets where consumer behaviors are still forming and complex to model using traditional parametric assumptions alone.

From a model performance perspective, it is essential to emphasize that the three analytical approaches used in this study are not directly comparable in terms of variance explanation. While pseudo-R^2^ measures provide a meaningful indication of explanatory power for the ordinal logistic regression (Nagelkerke R^2^ = 0.60), such metrics do not apply to ANN and decision tree models, which are designed for pattern recognition and classification rather than likelihood-based inference. In this context, the ANN’s moderate predictive accuracy and the CHAID tree’s clear segmentation structure should be interpreted as complementary strengths rather than competing explanatory benchmarks. The decision tree serves as a validation layer that corroborates the ANN’s identification of the central roles of knowledge and income, while enhancing interpretability for policy- and stakeholder-oriented applications. This layered modeling strategy aligns with recent recommendations in behavioral and food economics research, where machine learning methods are increasingly employed as exploratory and confirmatory tools alongside traditional inferential models, rather than as substitutes for them.

## 5. Conclusions

This study provides comprehensive insights into honey consumption patterns, consumer preferences, and willingness-to-pay (WTP) among Kosovar consumers, utilizing a combination of artificial neural networks (ANN), decision trees (CHAID), and ordinal logistic regression. These methods collectively identified knowledge about honey, income level, and trusted sources of recommendation (e.g., doctors, pharmacists) as the most significant predictors of consumer behavior and price sensitivity. Local honey was favored, and attributes such as botanical origin, geographical provenance, organic certification, and taste emerged as primary determinants in consumer evaluations. The consistency of results across methods strengthens the validity of these findings and provides robust evidence for stakeholders. While ANN offered predictive accuracy, the decision tree model provided interpretable segmentation, and the ordinal regression added statistical confirmation of variable effects. Together, these tools allowed for a nuanced understanding of how and why different consumer groups assign value to honey.

The findings of this study offer clear guidance for various stakeholders in the honey sector. For beekeepers and producers, transparent labeling that highlights botanical origin, geographical provenance, and organic certification is essential to build consumer trust and justify higher price points. Given the strong preference for direct purchases, producers should focus on local and direct-to-consumer channels. Policymakers and extension services can play a crucial role by investing in public education campaigns, particularly those leveraging the authority of doctors and pharmacists, to improve honey literacy and increase willingness to pay (WTP). Simultaneously, formalizing standards and supporting local quality assurance systems would enhance consumer confidence and rural income stability. For retailers and marketers, strategies that promote certified attributes and the health benefits of honey are likely to strengthen consumer loyalty. Moreover, the consumer segments identified through decision tree analysis can inform more effective, targeted marketing approaches, particularly by tailoring messages to income and knowledge levels.

Despite the strengths of a multi-method approach, this study faces several inherent limitations that should be considered in light of its exploratory and applied focus. The reliance on self-reported data may introduce minor social desirability bias; however, the anonymous and voluntary nature of the survey helps mitigate this risk. Additionally, although the sample size is robust, the use of an online distribution method may have slightly limited participation among older or less digitally engaged individuals, suggesting that future studies should include more offline respondents to improve representativeness. Lastly, while both the artificial neural network and decision tree models offered valuable insights, their lower accuracy in predicting responses in smaller WTP categories reflects a common challenge in real-world consumer research with class imbalance. Future research could address this by oversampling underrepresented groups or applying advanced balancing techniques to improve classification accuracy. Expanding the analysis to other regions or incorporating comparative case studies could also enhance the generalizability of the findings.

Beyond its empirical contribution, this study carries important implications for rural development and territorial policy in the Western Balkans. The results highlight that consumer trust and perceived authenticity are not only market signals but also foundations of rural resilience. In Kosovo’s context—where smallholder beekeeping remains fragmented and institutional support for certification and marketing is limited—enhancing transparency and quality communication could strengthen both rural incomes and community identity. By valorizing attributes such as origin, botanical type, and organic production, the honey sector can function as a lever for local value creation and territorial branding, positioning Kosovo within the broader European movement toward short food chains and sustainable consumption. Policymakers can therefore view the honey market as an entry point for broader rural diversification, linking consumer demand for trustworthy, local products with producer incentives for quality upgrading. Integrating these insights into rural development strategies, extension programs, and LEADER-type initiatives could foster cooperation among producers, municipalities, and consumers, ultimately contributing to a more embedded and inclusive rural economy.

Limitations: This study has some limitations. The use of convenience and online sampling resulted in an overrepresentation of younger, more educated, and urban respondents, introducing potential selection bias and limiting the generalizability of the findings to older adults, rural residents, and individuals with lower digital literacy. Additionally, the reliance on self-reported data may involve discrepancies between stated and actual behaviors, as well as recall bias and social desirability bias, which can affect the accuracy of responses. These factors should be considered when interpreting the results, and future studies would benefit from more representative sampling strategies and complementary objective data.

## Figures and Tables

**Table 1:** Socio-demographic variables of the survey respondents.

Variable	Categories	Frequency (%)
Gender	Female	39.8
	Male	60.2
Age	18–24 years	16.9
	25–34 years	40.4
	35–44 years	24.7
	45–54 years	9.3
	Above 55 years	8.7
Residence type	Rural	16.9
	Urban	83.1
Education	Middle school	7.4
	High school	28.4
	Bachelor	40.6
	Master	21.7
	Doctorate	2.0
Income	<200	2.0
	200–400	7.2
	400–600	15.7
	600–1000	23.3
	1000–1500	20.9
	Above 1500	31.0

**Table 2:** Variation in Honey Use from Infancy to Adulthood.

Category	0 kg	0.5–0.9 kg	1–2 kg	3–4 kg	5–6 kg	>6 kg	No Member in This Age	No Answer
Children 0–1 years (%)	18.1	14.9	2.8	1.0	0.4	0.4	12.7	49.7
Children 1–2 years (%)	12.3	19.5	7.0	2.0	0.4	0.2	12.5	46.1
Children 3–4 years (%)	8.9	21.7	10.1	3.2	0.4	0.8	11.7	43.1
Children 5–12 years (%)	6.8	21.5	15.3	5.2	1.4	1.4	10.9	37.6
Children 13–18 years (%)	5.8	17.9	16.5	5.4	1.8	1.8	9.7	41.2
Above 18 years (%)	4.0	19.5	28.4	14.5	3.6	6.6	2.8	20.7
Parents (%)	1.6	18.5	31.8	18.7	7.8	7.4	1.6	12.7
Grandparents (%)	3.6	14.3	21.5	11.5	4.8	4.2	9.9	30.2
Average	6.14	18.48	16.68	7.69	2.58	2.85	8.98	35.16

Notes: Honey consumption levels over 1-year period for all age groups. Results are given in percentage.

**Table 3:** Ordinal Logistic Regression Estimates for Willingness to Pay (WTP). Significance levels: *p* < 0.05 (*), *p* < 0.01 (**), *p* < 0.001 (***).

Variable	Estimate	Std. Error	*p*-Value	Significance
Gender	0.444	0.459	0.334	
Income	0.43	0.156	0.006	**
Knowledge = 2	1.319	1.162	0.257	-
Knowledge = 4	1.724	1.132	0.128	-
Information source = 1, 4	−7.522	2.837	0.008	**
Recommendation = 1	−7.591	1.534	0	***
Recommendation = 2	−6.569	1.458	0	***
Recommendation = 3	−5.784	1.418	0	***
Recommendation = 4	−7.604	1.541	0	***
Recommendation = 5	−3.984	1.604	0.013	*
Recommendation = 6	−9.214	2.586	0	***
Attributes = 1	−17.398	0.912	0	***
Attributes = 2	−17.147	0.895	0	***
Attributes = 3	−19.124	1.094	0	***
Attributes = 4	−16.379	1.057	0	***
Attributes = 5	−17.318	1.506	0	***
Attributes = 6	−18.042	1.068	0	***
Place = 1	−2.427	1.574	0.123	-

Knowledge = 2: Respondents with little knowledge about honey; Knowledge = 4: Respondents with good knowledge (reference category = very good knowledge). Info source = 1, 4: Information sourced from a combination of scientific articles and friends/family. Recommendation = 1–6: Main sources recommending honey products—1 = Doctor, 2 = Pharmacist, 3 = Family, 4 = Friends, 5 = Beekeeper, 6 = Other (reference = No recommendation). Attributes = 1–6: Consumer attributes prioritized when purchasing honey—1 = Geographical origin, 2 = Botanical origin (flower type), 3 = Price, 4 = Organic certification, 5 = Label, 6 = Taste (reference = Other attributes). Place = 1: Honey purchased in markets (reference category includes other places such as direct from the beekeeper, pharmacy, online, etc.).

## Data Availability

The original contributions presented in this study are included in the article. Further inquiries can be directed to the corresponding author.
